# A systematic review and meta-analysis of prevalence of *Escherichia coli* in foods of animal origin in Ethiopia

**DOI:** 10.1016/j.heliyon.2018.e00716

**Published:** 2018-08-06

**Authors:** Ayalew Assefa, Amare Bihon

**Affiliations:** a Sekota Dryland Agricultural Research Center, Sekota, Ethiopia; b Semera University, College of Veterinary Medicine, Semera, Ethiopia

**Keywords:** Infectious disease, Public health, Food safety

## Abstract

*Escherichia coli* is one of the causes of gastrointestinal diseases worldwide causing millions of illness annually. The occurrence of *Escherichia coli* in foods of animal origin in Ethiopia is arguably high due to many reasons like illegal slaughtering of animals in open fields, unhygienic slaughter practices in the abattoirs, and the risk of disease due to this organism is high because of a widespread tradition of raw meat consumption. The objective of this systematic review and meta-analysis was to pool estimates of the prevalence of the organism in different foods of animal origin which is the first of its kind in the country. The literature search was conducted to identify all published articles reporting the prevalence of *Escherichia coli* in foods of animal origin. From all screened articles, 30 studies were eligible for final systematic review and meta-analysis. Because substantial heterogeneity was expected, random-effects meta-analyses were carried out to pool the prevalence of the organism from different foods of animal origin. The result indicated that between-study variability was high (τ2 = 0.00; heterogeneity I^2^ = 96.77% with Heterogeneity chi-square = 1298.92, a degree of freedom = 42 and a P-value of = 0.001) with the overall random pooled prevalence of 15% (95% CI = 13%–17%) in foods of animal origin. The result of meta-regression showed diagnosis method used, sample size and study year had contributed significantly to the heterogeneity of studies. This systematic review and meta-analysis showed the level of contamination of foods of animal origin in Ethiopia is high indicating the need for immediate planning of mitigation strategies and detection methods to reduce its level and impact throughout the country.

## 1 Introduction

Foodborne pathogens including *Escherichia coli, Listeria monocytogenes, Salmonella species, Staphylococcus aureus,* and many more other organisms are the leading causes of foodborne illness and death in the world (Ajayi et al., 2011). The reason for the increased risk can be attributed to many reasons which include but not limited to changes in eating habits, mass catering, complex and lengthy food supply procedures with increased international movement and poor hygiene practices. Foodborne zoonotic diseases often occur due to the consumption of contaminated food-stuffs especially from animal products such as meat and milk (Bevilacqua et al., 2017; Lopez-Campos et al., 2012).

*Escherichia coli* is characterized as a gram-negative, rod-shaped bacterium belonging to the family Enterobacteriaceae with five virulence groups, including; enteroaggregative *Escherichia coli* (EAEC), enterohemorrhagic *Escherichia coli* (EHEC), enteroinvasive *Escherichia coli* (EIEC), enteropathogenic *Escherichia coli* (EPEC), and enterotoxigenic (ETEC). *Escherichia coli* O157: H7, one of the virulent strain under the species *Escherichia coli* is a leading cause of hemorrhagic colitis, hemolytic-uremic syndrome (HUS) and thrombotic thrombocytopenic purpura (TTP) in man (Cobbaut et al., 2009; Earley and Leonard, 2006). This strain is considered as one of the severe foodborne disease-causing organism globally. The risk and consequences of the organism are severe in developing countries due to different reasons that are attributable to poor livelihood in general. The food types most commonly associated with outbreaks of food poisoning due to *Escherichia coli* O157 are mostly of bovine origin, in particular, beef and beef burgers and unpasteurized milk (Hussein et al., 2001; Pennington, 2010; Söderlund, 2015). However, it has been increasingly recognized that fresh vegetables and fruits other than beef or beef-product can be the sources of Shiga toxin-producing *Escherichia coli* (STEC) infection (Cheol et al., 2013; Gomes et al., 2016; Karmali et al., 2010).

Gastroenteritis due to foodborne disease is one of the most common illnesses in Ethiopia, and it is a leading cause of death among people of all ages in the country (Ayana et al., 2015). Consumption of raw beef is commonly practiced in Ethiopia. Generally, illegal slaughtering of animals in open fields, unhygienic slaughter practices in the abattoirs, and wide-spread tradition of consumption of raw meat (Kitfo dulet and Kurt) are potential risk factors in the country. Given that ruminants are natural reservoirs of Shiga toxin-producing *Escherichia coli* strains, higher prevalence is common in areas where fecal contamination is continuous, namely, farms, transportation trucks used to deliver animals to slaughtering houses, and slaughtering halls (Bekele et al., 2014; Beyi et al., 2017; Disassa et al., 2017).

A handful of studies have been conducted in Ethiopia that reports the occurrence level of *Escherichia coli* in foods of animal origin mostly in meat and milk in recent years (Atnafie et al., 2017; Dulo et al., 2015; Mengistu et al., 2017; Mersha et al., 2010; Taye, 2013). Most of the studies were conducted in central Ethiopia due to extensive animal farming practices in those areas. There are also studies throughout the country that estimate the prevalence of the organism in foods of animal origin. National level estimation and quantification of *Escherichia coli* occurrence can help responsible bodies for the prevention and control of its occurrence in foods before reaching end users ultimately reducing its impact. To the best of our knowledge, this review is the first of its kind in estimating the pooled prevalence of *Escherichia coli* in foods of animal origin in Ethiopia. The objective of the study was to pool estimates of proportions of occurrence of *Escherichia* coli in foods of animal origin to have a national estimate of the occurrence level of the organism.

## 2 Methodology

### 2.1 Literature search strategy

**Fig. 1:**
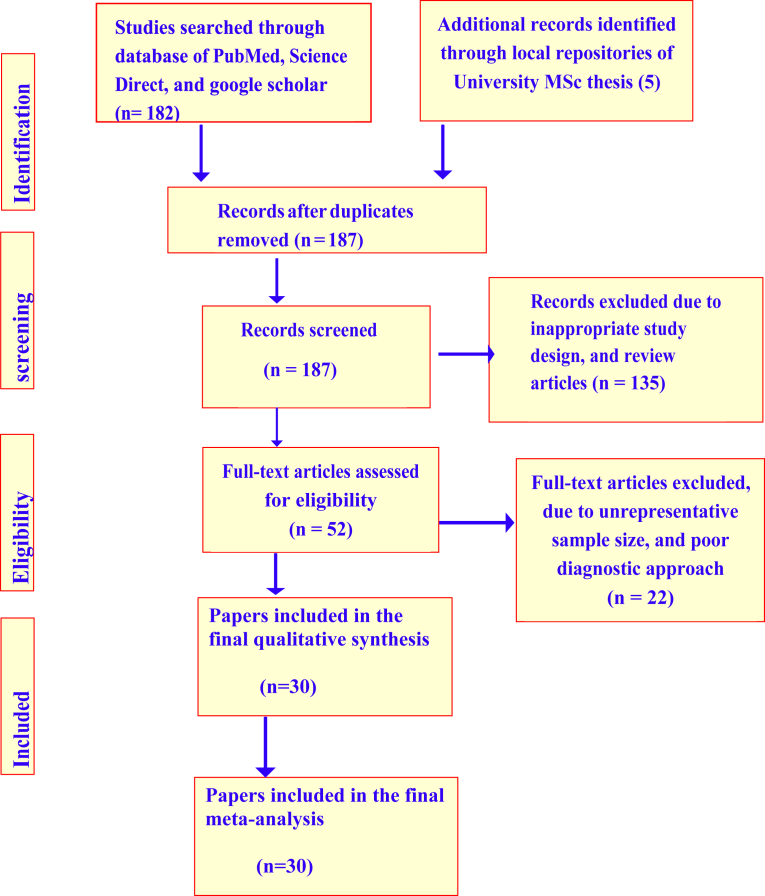
Flowchart of literature search and inclusion/exclusion process.

The literature search was conducted to identify all published studies reporting the prevalence of *Escherichia coli* in foods of animal origin (meat and milk). It was conducted in electronic databases of PubMed, google scholar, African Journals Online, EMBase, and Scopus from November 2017 to May 2018. The specific search medical subject heading (MeSH) terms include “*Escherichia coli* and Ethiopia”, “*Escherichia coli* prevalence and Ethiopia”, “*Escherichia coli* O17: H7 and Ethiopia”, “*Escherichia coli* and meat in Ethiopia”, “*Escherichia coli* in milk in Ethiopia”, “*Escherichia coli*” AND “Ethiopia” AND “prevalence”. Based on the intensive literature search, a total of 187 pieces of literature that report the prevalence of *Escherichia coli* in Ethiopia were retrieved. However, only 30 reports on the prevalence of *Escherichia coli* in meat, milk, and related working environmental samples made it to the final meta-analysis procedure. Study screening strategy and exclusion reasons are presented in Fig. 1.

### 2.2 Eligibility criteria and data extraction procedure

All Articles that report the prevalence of *Escherichia coli* in meat and milk in Ethiopia were downloaded and added to Mendeley reference manager. Duplicates were rigorously checked and removed. Quality criteria were developed before starting the review of full papers. Inclusion/exclusion criteria were defined regarding the relevance of the articles to the research questions of interest. The inclusion criteria include an observational study that reports prevalence of the diseases, published article or MSc thesis in University online repositories, reporting the prevalence of the organism only in foods of animal origin from 2000 to 2017, diagnostic methods that employed one of the diagnostic approaches (culture, Latex agglutination, and molecular methods). Articles that met the above criteria were considered for the final meta-analysis and systematic review. Titles were checked twice in both excluded and included databases of the Mendeley reference manager before the start of the data extraction process to avoid missing a valuable report independently. Those papers considered relevant were retained, and their results were extracted to a pre-prepared data extraction excel sheet. Data extracted from valuable papers include study area, study year, sample size, number of positives, food type examined, diagnosis method used, author's name, article title, and year of publication.

### 2.3 Statistical analysis method

Statistical analyses were done using Stata 14 (StataCorp. 2015. Stata Statistical Software: Release 14. College Station, TX). A simple summary of reports with the prevalence of *Escherichia coli* was done by descriptive statistics. Meta-analysis of prevalence data was analyzed, pooled the estimates and the 95% confidence intervals. Due to the natures of studies, substantial heterogeneity was expected, and a random-effects meta-analysis was done with the estimate of heterogeneity being taken from an inverse-variance model (DerSimonian and Laird, 1986). Publication bias was assessed using the Eggers test, as well as visual inspection of the funnel plot. Between-study heterogeneity was assessed using Higgin's *I*^*2*^ and Cochran's Q method. *I*^*2*^ values of 25%, 50%, and 75% were considered low, moderate and high heterogeneity, respectively (Higgins and Thompson, 2002). Subgroup analysis was also conducted by food type sampled, the location of study and diagnosis method used. Meta-regression was used to investigate factors potentially contributing to the between-study heterogeneity. Univariable analysis was done for each selected variable included. Sample size as a continuous variable and categorized variable, study year as a categorized variable, and diagnosis method was used in the final meta-regression model.

## 3 Results

### 3.1 Descriptive results of eligible studies

From all screened studies, 30 articles were eligible for the final systematic review and meta-analysis. Literature was heterogeneous, had inappropriate study designs, unrepresentative sample size, and unreliable diagnostic methods. This diversity, together with the lack of data on other required variables, reduced our datasets substantially. Descriptive summary statistics were calculated to determine the total number of sampled foods and the range of prevalence estimates in different foods. Accordingly, the overall apparent prevalence in all studies estimated 18.1% in all samples examined. Few studies reported the pathogenic strain of *Escherichia coli* O 157: H7 either with molecular examination or latex agglutination tests, while most of the studies reported at the species level (*Escherichia coli*) by culture and biochemical tests. A detailed summary of the studies can be found in Table 1.

**Table 1:** Descriptive statistics of included studies in the final systematic review and meta-analysis.

Study (references)	Year	SS	NP	AP (%)	Region	Diagnosis method used	Strain isolated	Sample type
Zuleka et al. (2016)	2016	288	30	21.74	Southern Ethiopia	Culture and biochemical test	*-*	Milk
Wubet et al. (2014)	2014	84	26	30.95	Western Ethiopia	Culture and biochemical test	*-*	Milk
Taye (2013)	2013	113	35	30.97	Eastern Ethiopia	Culture and biochemical test	*-*	Beef
Tassew et al. (2010)	2010	165	44	26.67	Western Ethiopia	Culture and biochemical test	*-*	Beef
Reta et al. (2016)	2016	120	70	58.33	Southern Ethiopia	Culture and biochemical test	*-*	Milk
Mohammed et al. (2014)	2014	384	61	15.89	Eastern Ethiopia	Culture and biochemical test	*-*	Beef
Messele et al. (2017)	2017	73	4	5.48	Central Ethiopia	Culture and biochemical test	*-*	Beef
Messele et al. (2017)	2017	73	27	36.99	Central Ethiopia	Culture and biochemical test	*-*	Chicken
Messele et al. (2017)	2017	73	17	23.29	Central Ethiopia	Culture and biochemical test	*-*	Mutton
Messele et al. (2017)	2017	73	15	20.55	Central Ethiopia	Culture and biochemical test	*-*	Chevon
Mersha et al. (2010)	2010	120	8	6.67	Central Ethiopia	Molecular diagnosis (PCR)	*Escherichia coli O157:H7*	Chevon
Mersha et al. (2010)	2010	224	21	9.38	Central Ethiopia	Molecular diagnosis (PCR)	*Escherichia coli O157:H7*	Mutton
Mersha et al. (2010)	2010	344	13	3.78	Central Ethiopia	Molecular diagnosis (PCR)	*Escherichia coli O157:H7*	Environmental samples
Mengistu et al. (2017)	2017	30	7	23.33	Eastern Ethiopia	Culture and biochemical test	*-*	Environmental samples
Mengistu et al. (2017)	2017	290	36	12.41	Eastern Ethiopia	Culture and biochemical test	*-*	Beef
Melkamsew et al. (2012)	2012	450	51	11.33	Central Ethiopia	Culture and biochemical test	*-*	Milk
Hilali et al. (2016)	2016	19	7	36.84	Northern Ethiopia	Culture and biochemical test	*-*	Milk
Hiko et al. (2015)	2015	119	35	29.41	Central Ethiopia	Culture and biochemical test	*-*	Beef
Hiko et al. (2008)	2008	243	6	2.47	Central Ethiopia	Latex agglutination test	*Escherichia coli O157:H7*	Mutton
Hiko et al. (2008)	2008	245	5	2.04	Central Ethiopia	Latex agglutination test	*Escherichia coli O157:H7*	Chevon
Hiko et al. (2008)	2008	250	20	8.00	Central Ethiopia	Latex agglutination test	*Escherichia coli O157:H7*	Beef
Haileselassie et al. (2013)	2013	33	9	27.27	Northern Ethiopia	Culture and biochemical test	*-*	Beef
Haile et al. (2017)	2017	150	14	9.33	Western Ethiopia	Latex agglutination test	*Escherichia coli O157:H7*	Beef
Haile et al. (2017)	2017	150	11	7.33	Western Ethiopia	Latex agglutination test	*Escherichia coli O157:H7*	Environmental samples
Hadush et al. (2008)	2014	69	15	21.74	Northern Ethiopia	Culture and biochemical test	*-*	Milk
Garedew et al. (2012)	2012	54	16	29.63	Northern Ethiopia	Culture and biochemical test	*-*	Milk
Dulo et al. (2015)	2014	235	6	2.55	Eastern Ethiopia	Latex agglutination test	*Escherichia coli O157:H7*	Chevon
Disassa et al. (2017)	2017	380	129	33.95	Western Ethiopia	Culture and biochemical test	*-*	Milk
Desta et al. (2016)	2016	106	49	46.23	Central Ethiopia	Culture and biochemical test	*-*	Milk
Desta et al. (2011)	2011	106	21	19.81	Central Ethiopia	Culture and biochemical test	*-*	Milk
Demme and Shimeles (2015)	2015	102	19	18.63	Central Ethiopia	Culture and biochemical test	*-*	Milk
Beyi et al. (2017)	2017	195	5	2.56	Central Ethiopia	Latex agglutination test	*Escherichia coli O157:H7*	Beef
Beyi et al. (2017)	2017	330	4	1.21	Central Ethiopia	Latex agglutination test	*Escherichia coli O157:H7*	Environmental samples
Bekele et al. (2014)	2014	384	39	10.16	Central Ethiopia	Latex agglutination test	*Escherichia coli O157:H7*	Beef
Balcha et al. (2014)	2014	80	50	62.50	Northern Ethiopia	Culture and biochemical test	*Escherichia coli*	Beef
Atnafie et al. (2017)	2017	300	8	2.67	Southern Ethiopia	Latex agglutination test	*Escherichia coli O157:H7*	Beef
Atnafie et al. (2017)	2017	330	7	2.12	Southern Ethiopia	Latex agglutination test	*Escherichia coli O157:H7*	Environmental samples
Amenu et al. (2014)	2014	53	11	20.75	Southern Ethiopia	Culture and biochemical test	*-*	Milk
Adugna et al. (2013)	2013	74	7	9.46	Southern Ethiopia	Culture and biochemical test	*-*	Milk
Abebe et al. (2014)	2014	192	43	22.40	Northern Ethiopia	Culture and biochemical test	*-*	Milk
Abebe et al. (2014)	2014	192	41	21.35	Northern Ethiopia	Culture and biochemical test	*Escherichia coli*	Beef
Abdissa et al. (2017)	2017	1235	6	0.49	Central Ethiopia	Latex agglutination test	*Escherichia coli O157:H7*	Beef
Abdissa et al. (2017)	2017	1247	10	0.80	Central Ethiopia	Latex agglutination test	*Escherichia coli O157:H7*	Environmental samples

SS sample size, NP number of positives, AP apparent prevalence.

### 3.2 Meta-analysis

**Fig. 2:**
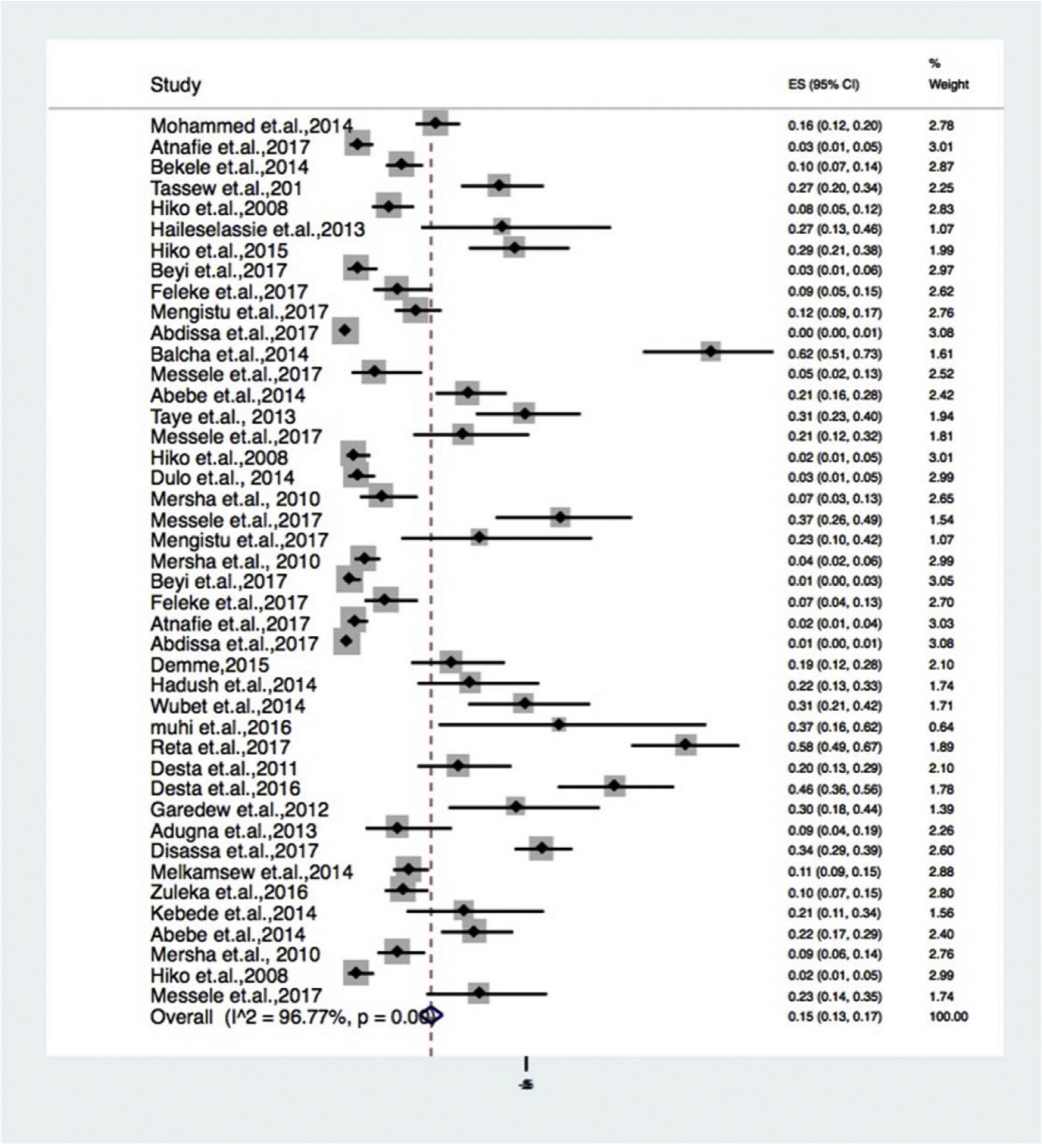
Forest plot on *Escherichia coli* in foods of animal origin prevalence estimates in Ethiopia.

Due to the expected variation between studies, random-effects meta-analyses were carried out using the total sample size and number of positives (effect size and standard error of the effect size). The meta-analysis indicated that between-study variability was high (τ2 = 0.001; heterogeneity I^2^ = 96.77% with Heterogeneity chi-square = 1298.92, degree of freedom = 42 and a P value of 0.001). Individual study prevalence estimates ranged from 0% to 62% with the overall random pooled prevalence of 15% (95% CI = 13%–17%). Studies weighted approximately equal with weights on individual studies ranging from 1.07% to 3.08% due to high heterogeneity between studies. Fig. 2 presents the Forest Plot derived from the meta-analysis.

### 3.3 Subgroup meta-analysis

**Fig. 3:**
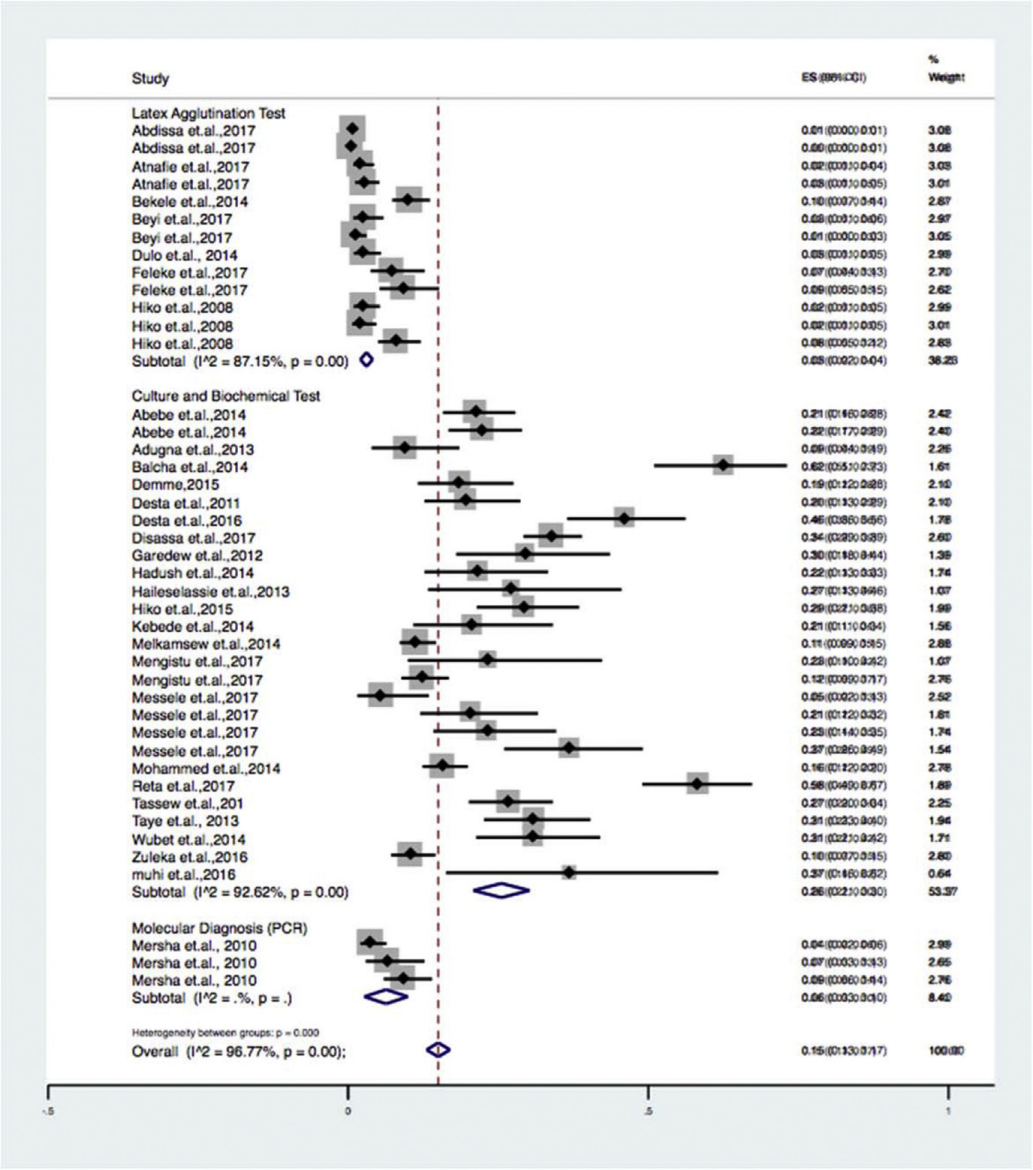
Forest plot of sub group analysis by diagnosis method used on *Escherichia coli* in foods of animal origin prevalence estimates in Ethiopia.

**Fig. 4:**
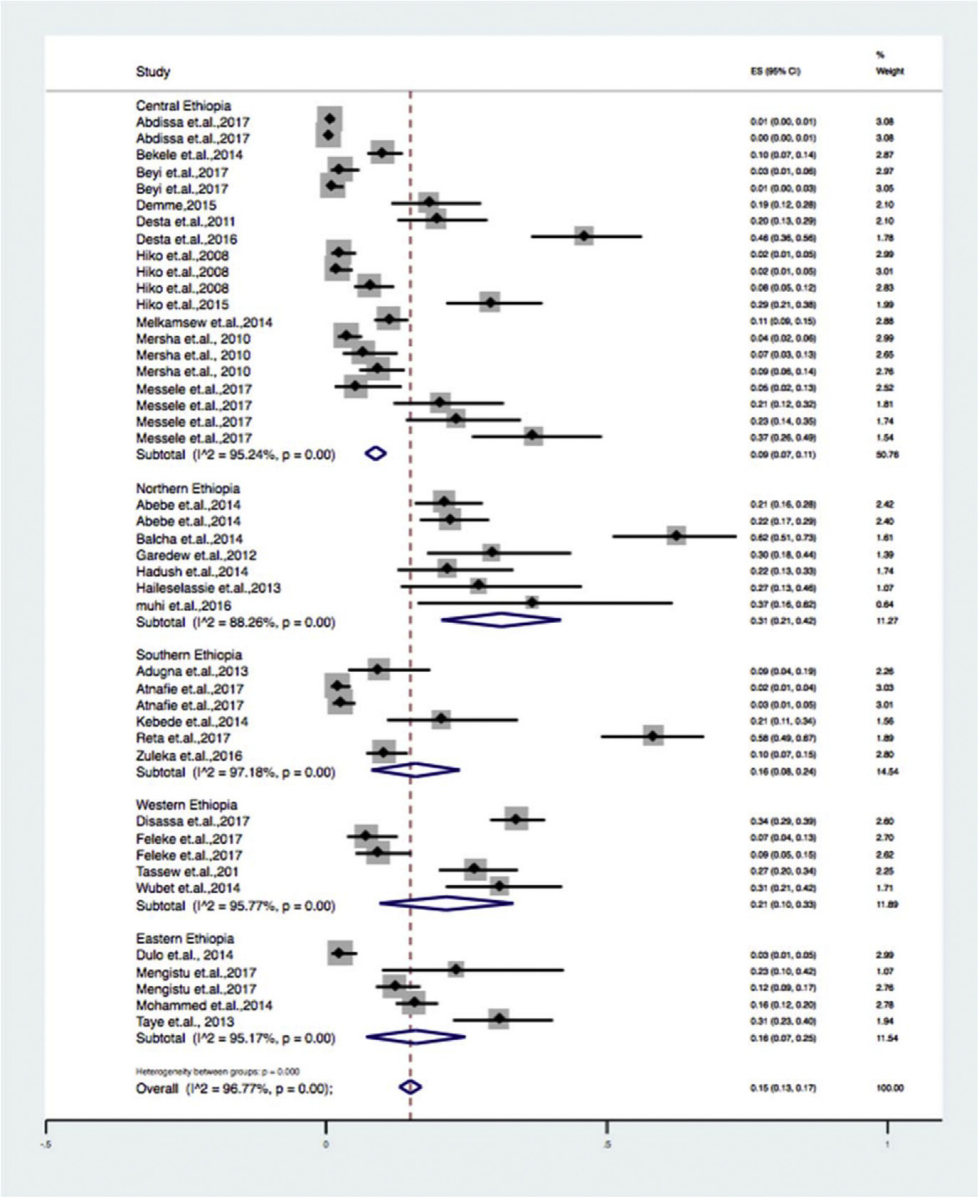
Forest plot of sub group analysis by study location on *Escherichia coli* in foods of animal origin prevalence estimates in Ethiopia.

**Fig. 5:**
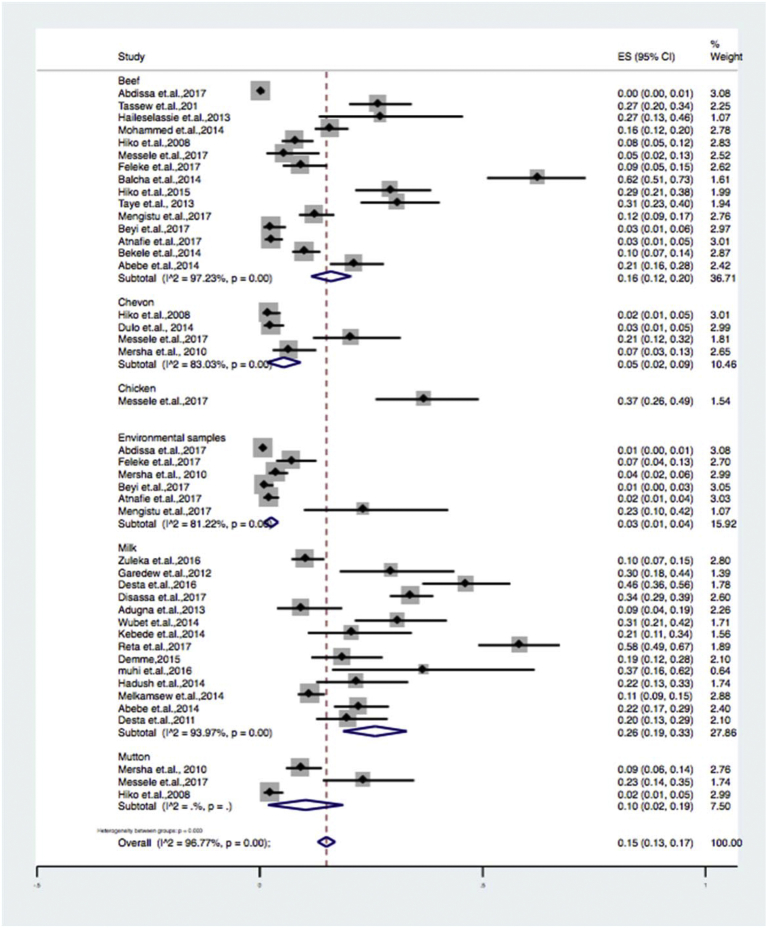
Forest plot of sub group analysis by sample type examined on *Escherichia coli* in foods of animal origin prevalence estimates in Ethiopia.

**Fig. 6:**
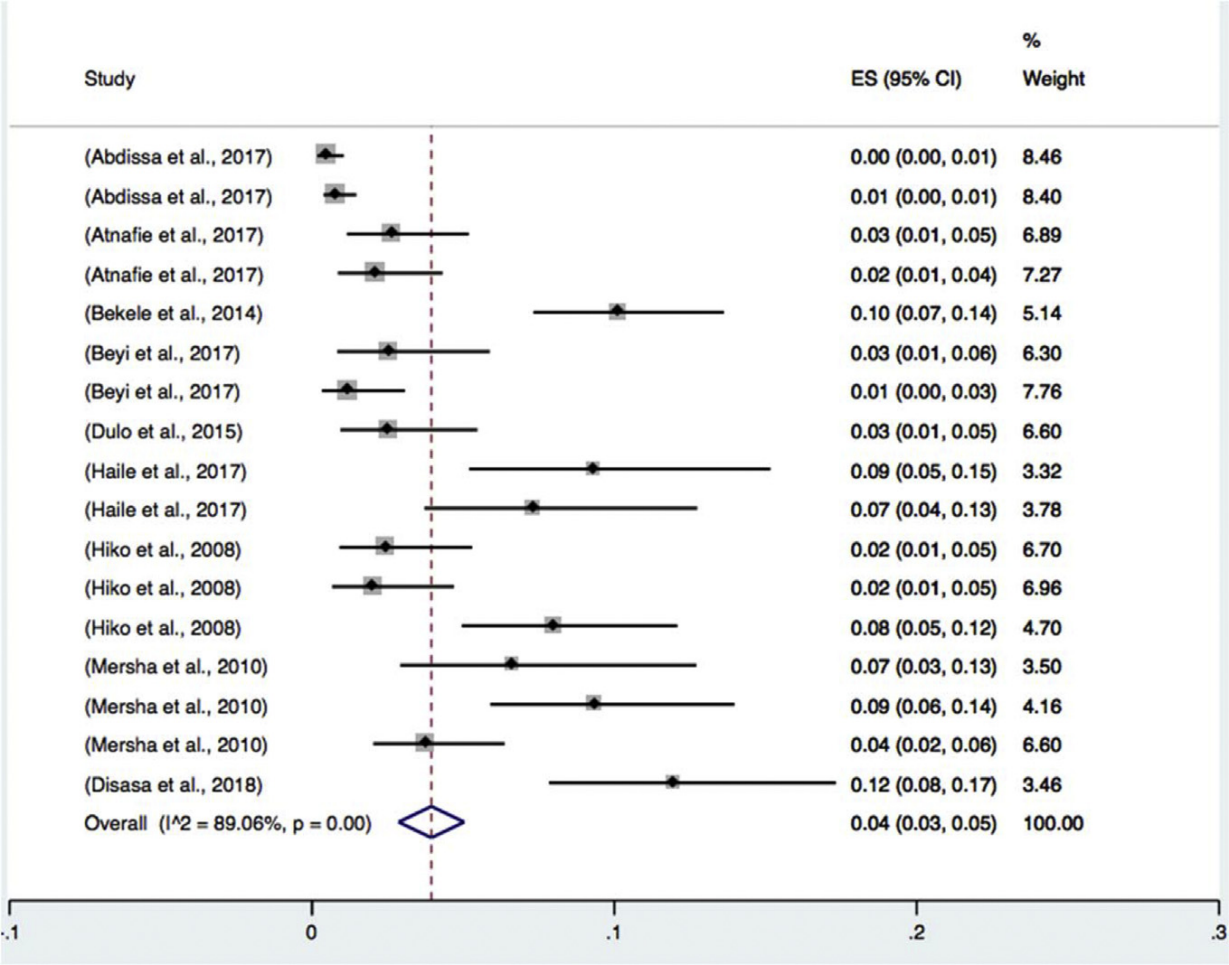
Forest plot on *Escherichia coli* O157 in foods of animal origin prevalence estimates in Ethiopia.

Subgroup analyses were done for sample types (Table 2) (beef, milk, mutton, chevon, chicken and environmental swabs), study locations (Table 3) (eastern, central, northern, southern and western Ethiopia), and diagnosis methods used (Table 4) (culture and biochemical method, latex agglutination and molecular techniques). Results of the subgroup analysis are depicted in Figs. 3, 4, and 5 respectively. We also performed a subgroup analysis for the prevalence of pathogenic of *Escherichia coli* O157: H7. The meta-analysis indicated that between-study variability was high (τ2 = 0.01; I^2^ = 89.06% with Cochran's Q statistics = 146.28, at a degree of freedom = 16 with a P value of = 0.01). Individual study prevalence estimates ranged from 0% to 10% with the overall random pooled prevalence of 4% (95% CI = 3%–5%). Fig. 6 presents the Forest plot derived from the meta-analysis of the pathogenic strain.

**Table 2:** Subgroup analysis for comparison of prevalence of *Escherichia coli* in different sample types examined.

Sample type	Prevalence (95%CI)	I^2^	Q	Heterogeneity test	
				DF	*P*
Beef	6 (12–20)	97.2%	506.05	14	0.00
Chevon	5 (2–9)	83.0%	17.67	3	0.00
Chicken	37 (26–49)	-	-	0	-
Environmental sample	3 (1–4)	81.22%	26.63	5	0.00
Milk	26 (0.19–0.33)	93.97%	215.63	13	0.00
Mutton	10 (2–19)	-	-	2	-
Overall	15 (13–17)	96.7%	1298.92	42	0.00

**Table 3:** Subgroup analysis for comparison of prevalence of *Escherichia coli* in different geographical locations across Ethiopia.

Region	Prevalence% (95%CI)	I^2^	Q	Heterogeneity test	
				DF	*P*
Eastern Ethiopia	16 (7–25)	95.1%	82.89	4	0.00
Southern Ethiopia	16 (8–24)	97.1%	177.34	5	0.00
Central Ethiopia	9 (7–11)	95.2%	399.14	19	0.00
Western Ethiopia	21 (10–33)	95.77%	94.45	4	0.00
Northern Ethiopia	0.31 (2–42)	88.26%	51.10	6	0.00
Overall	0.15 (13–17)	96.7%	1298.92	42	0.00

**Table 4:** Subgroup analysis for comparison of prevalence of *Escherichia coli* in by diagnosis method used.

Diagnosis method	Prevalence (95%CI)	I^2^	Q	Heterogeneity test	
				DF	*P*
Culture and biochemical tests	26 (0.21–0.30)	92.6%	352.16	26	0.00
Latex agglutination	3 (0.02–0.04)	87.15%	93.35	12	0.00
Molecular diagnosis	6 (3–10)	.	.	2	.
Overall	15 (13–17)	96.7%	1298.92	42	0.00

### 3.4 Meta-regression

**Fig. 7:**
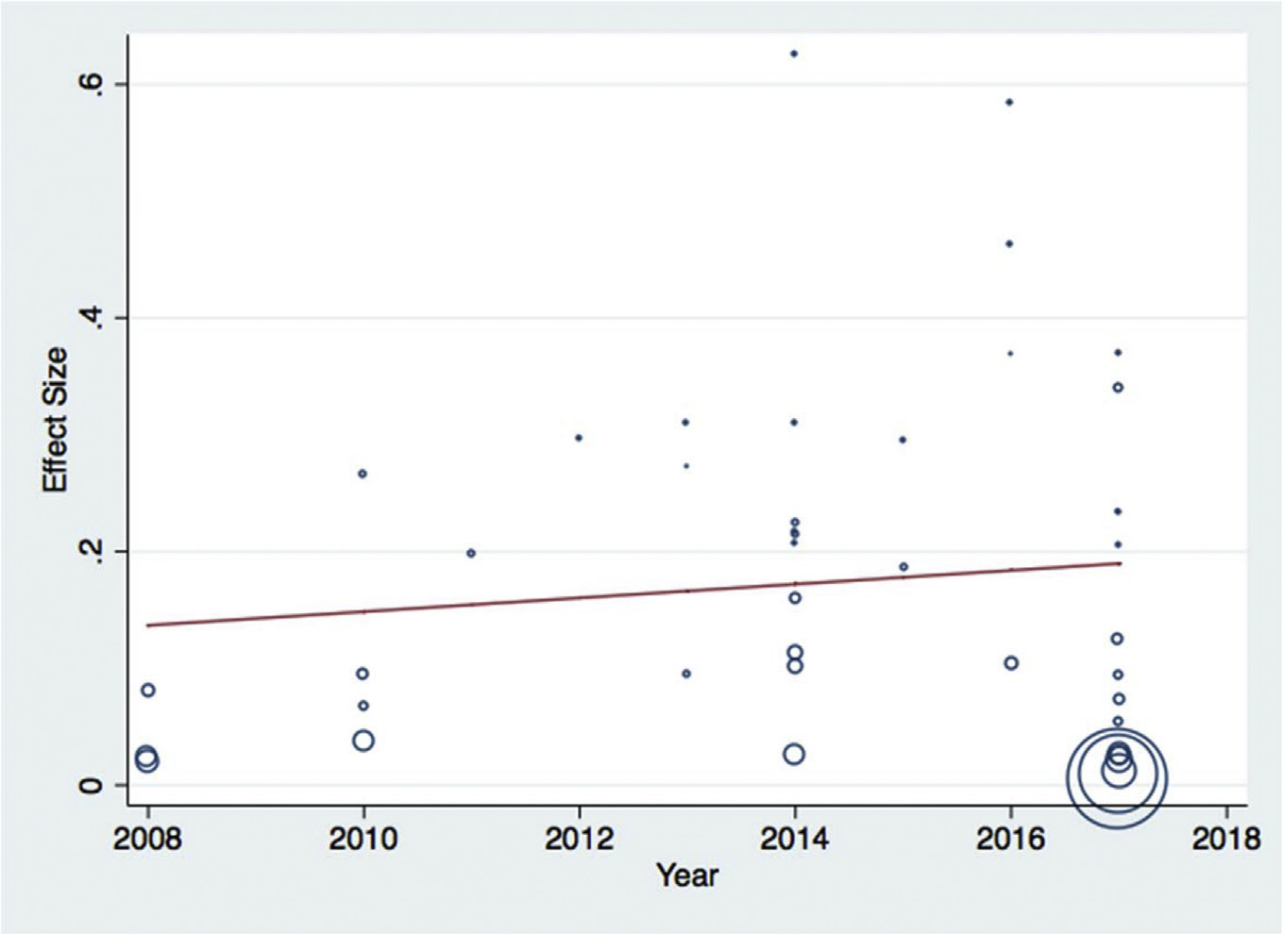
Meta-regression plot of study year versus prevalence of *Escherichia coli* in foods of animal origin in Ethiopia.

**Fig. 8:**
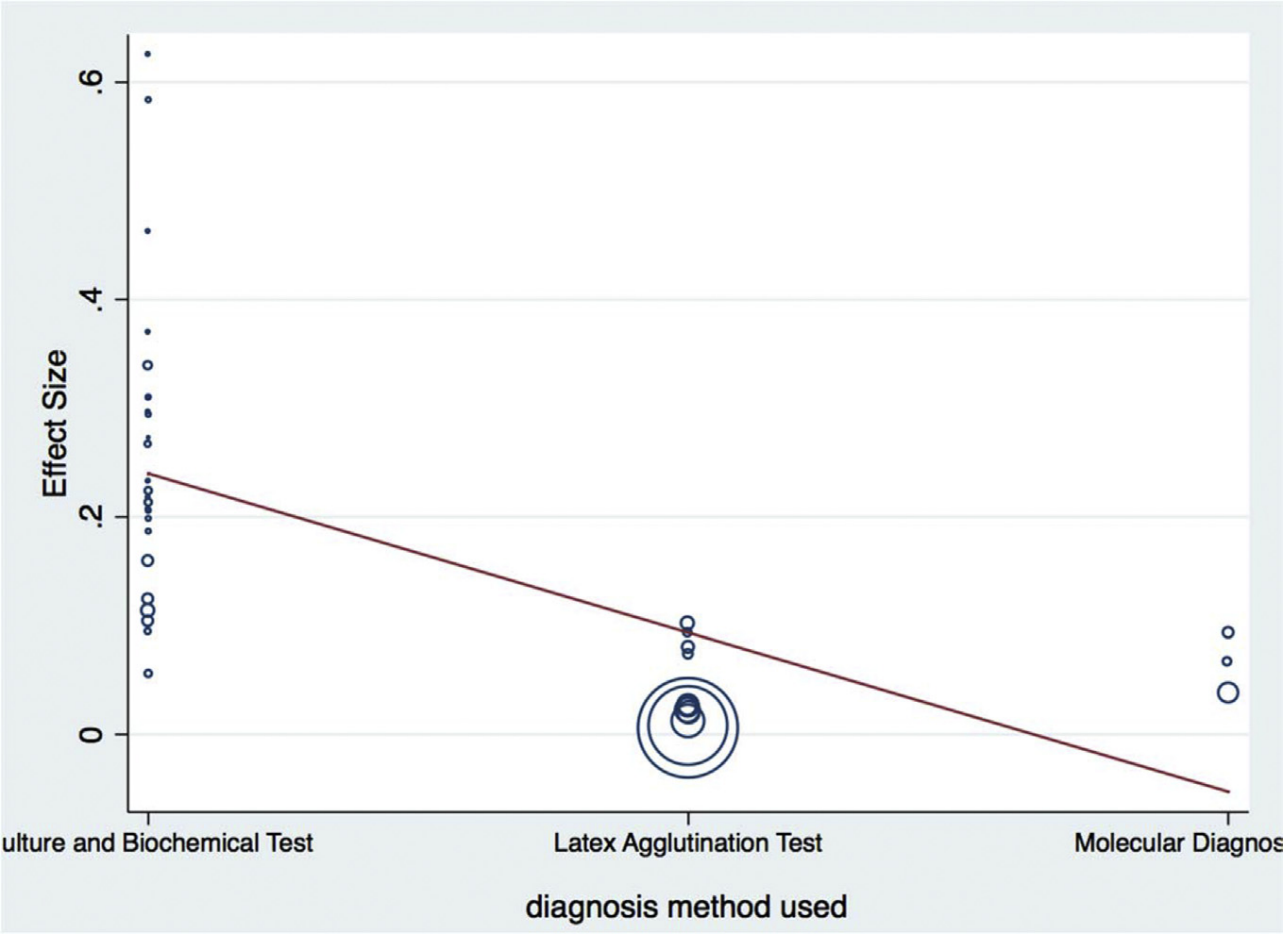
Meta-regression plot of diagnosis method used versus prevalence of *Escherichia coli* in foods of animal origin in Ethiopia.

**Fig. 9:**
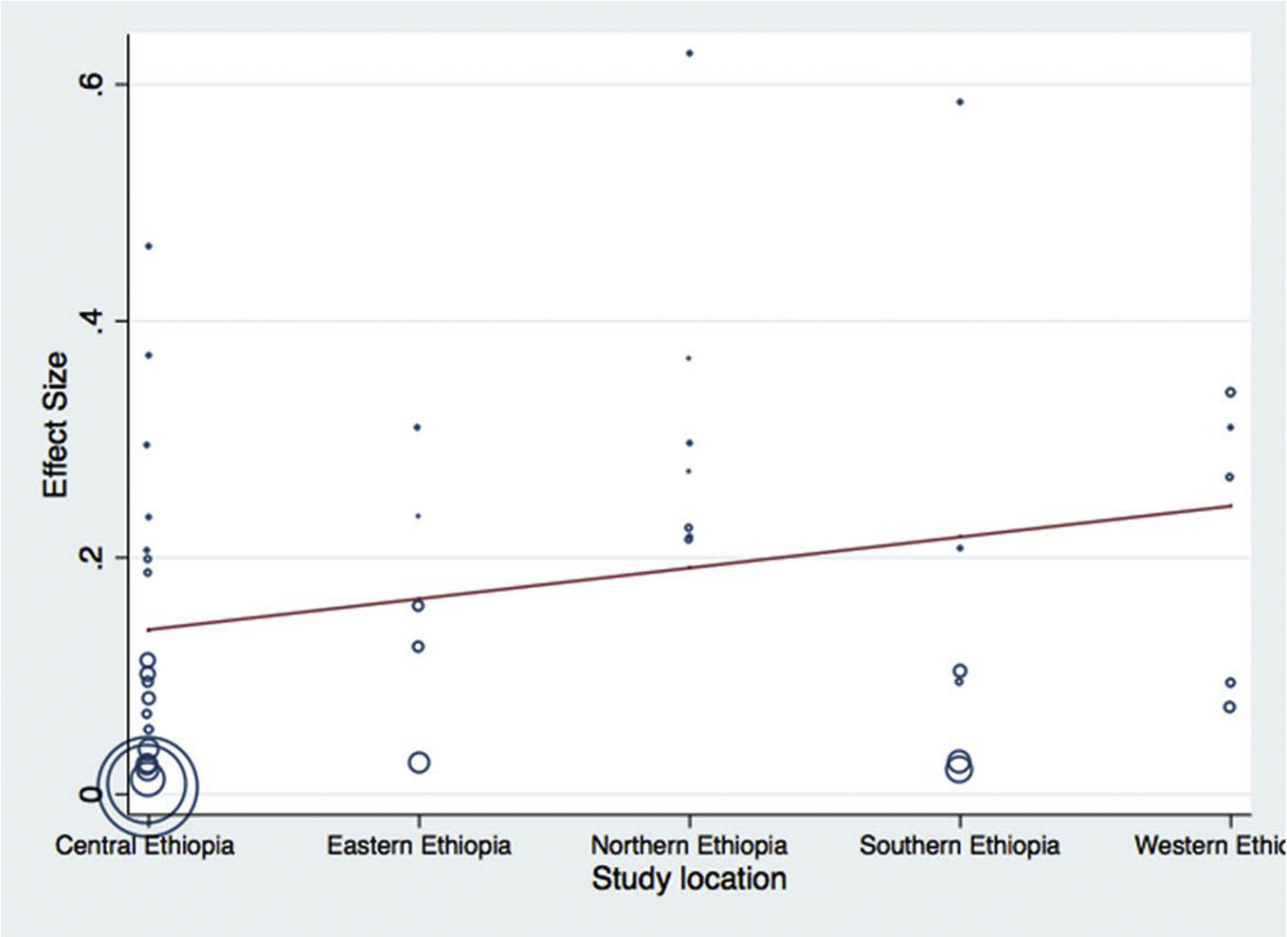
Meta-regression plot of study location versus prevalence of *Escherichia coli* in foods of animal origin in Ethiopia.

**Fig. 10:**
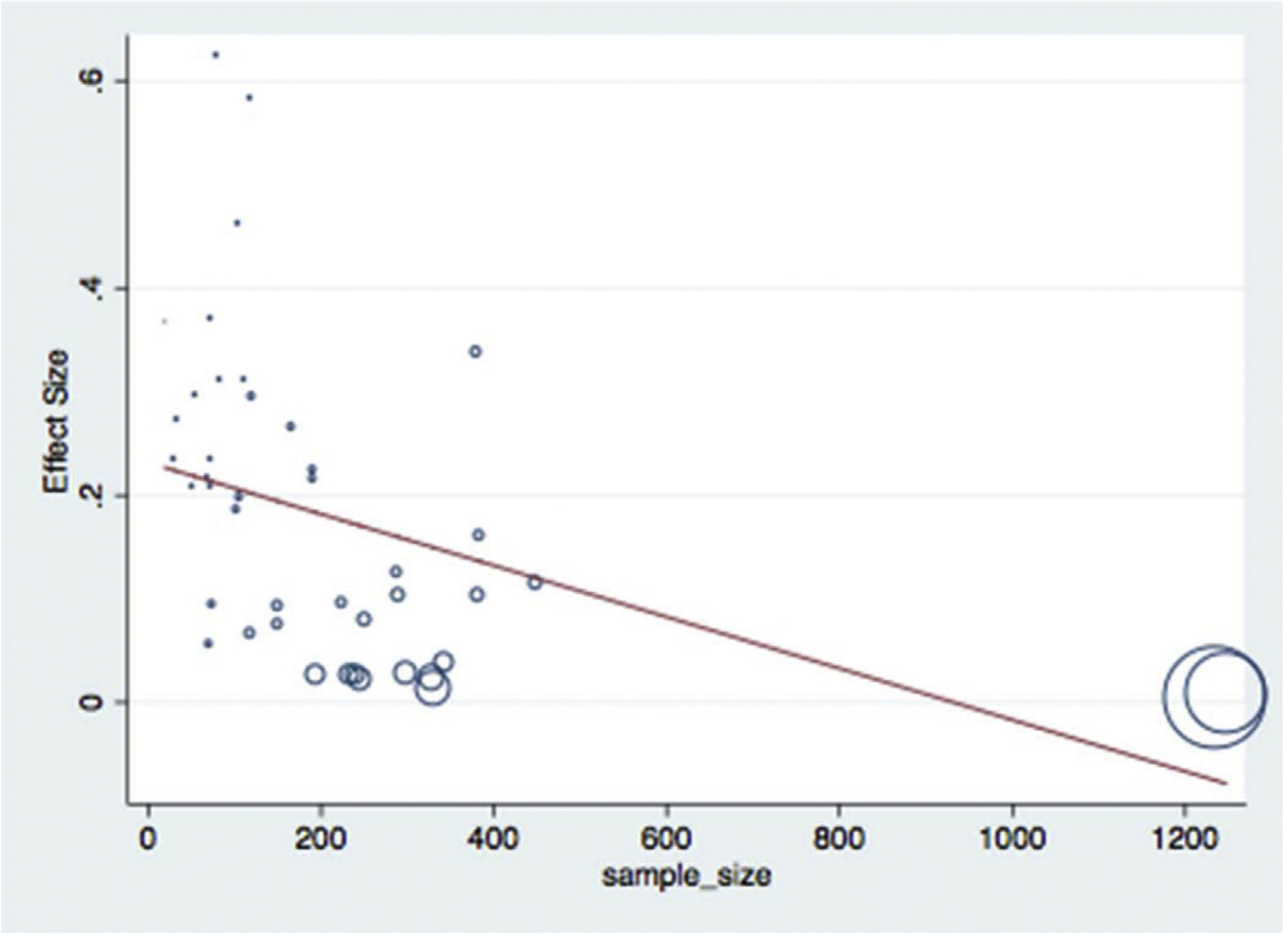
Meta-regression plot of sample size versus prevalence of *Escherichia coli* in foods of animal origin in Ethiopia.

Meta-regression analysis was done for each variable included in the study separately. The variables included were sample size as a continuous and categorized variable, study location, sample types examined, study year as a categorical variable, and diagnosis method used. Continuous variable (sample size) was subjected to assessment to see a linear relationship between the independent effect size. Those variables with a p-values <0.1 were used in the multivariable meta-regression analysis. Categorized sample size, study year as a categorical variable, and diagnosis method used had significant value and retained in the final multivariable analysis. Results of final multivariable meta-regression are summarized in Table 5 and in Figs. 7, 8, 9, and 10.

**Table 5:** Final multivariable meta regression model.

Variables	Coefficient	P-value	95% CI
**Diagnosis method used**			
Culture and biochemical tests	Reference		
Latex agglutination	−0.146	0.002	−0.23 to −0.06
Molecular diagnosis	−0.137	0.064	−0.28 to 0.008
**Sample size category**			
Sample size less than 150	Reference		
Sample size between 151 and 300	−0.087	0.044	−0.17 to −0.002
Sample size greater than 300	−0.084	0.075	−0.17 to 0.009
**Study year category**			
Study year 2008–2013	Reference		
Study year 2014–2016	0.05	0.278	−0.043 to 0.15
Study year 2017	−0.016	0.737	−0.11 to 0.08
**Constant**	**0.26**	**0.000**	**0.18–0.34**

### 3.5 Publication bias and small study effect assessment

**Fig. 11:**
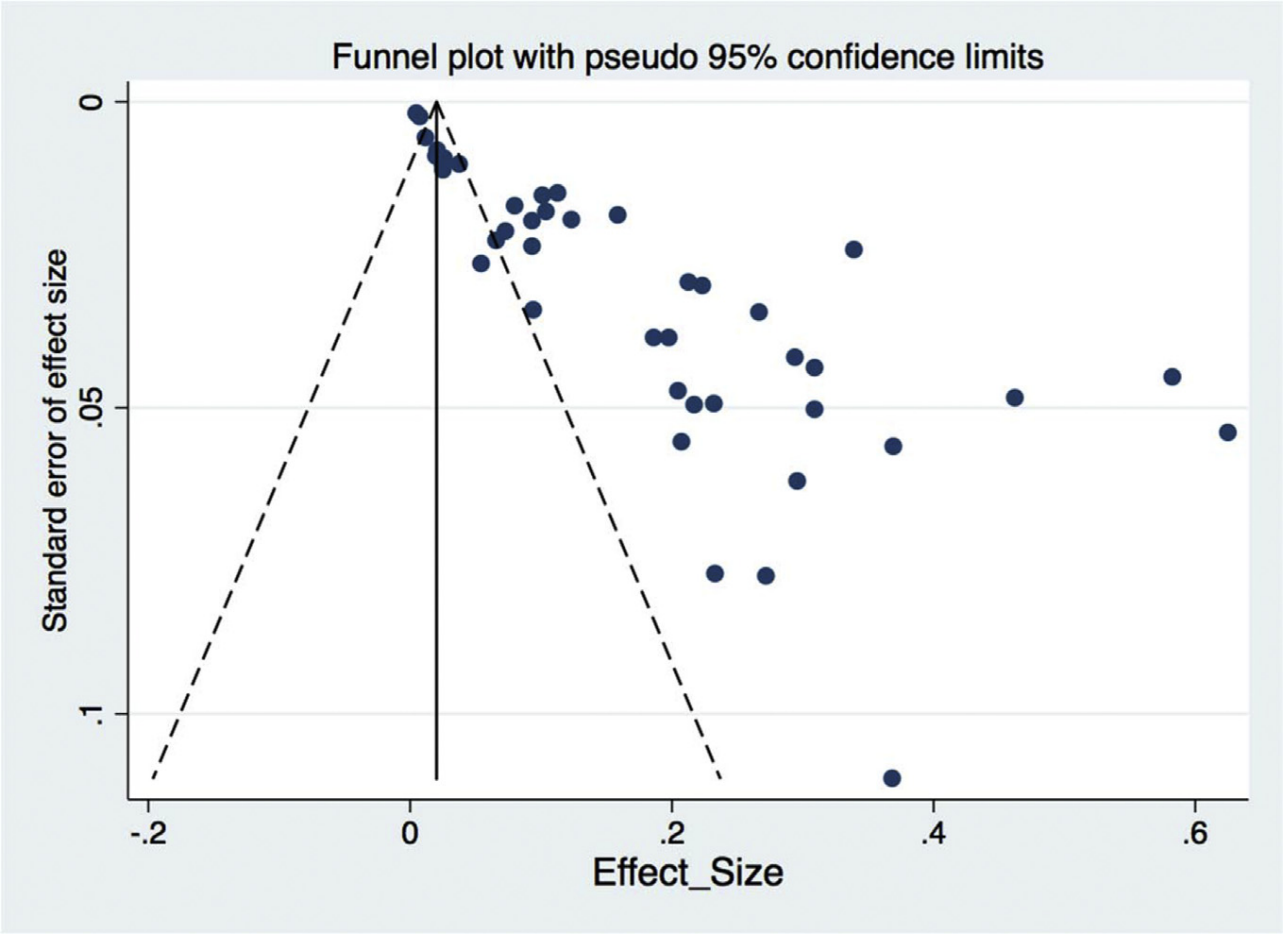
Funnel plot that assesses publication bias.

Even though assessing publication bias may not be applicable in situations like this because Begg's and Egger's test become unsuitable due to an association between the effect size and its standard error, we assessed bias and small study effects by funnel plot observation and Begg's and Egger's test for small-study effects. The result of effect estimates against its standard error showed that there was a publication bias with a p-value of 0.001. (see Table 6 and Fig. 11 for Egger's test for small-study effects and funnel plot assessment respectively).

**Table 6:** Eggers test for publication bias assessment.

Standard effect	Coefficient	t-value	p-value	95%CI
Slope	−0.01	−2.18	0.035	−0.02 to −0.00072
Bias	5.89	11.32	0.001	4.84–6.94

## 4 Discussion

To the best of our knowledge, this is the first systematic review and meta-analyses on the status of *Escherichia coli* in foods of animal origin in Ethiopia. The review process was limited to foods of animal origin to minimize heterogeneity between studies and absence of enough studies in other foodstuffs. This report was from the analysis of data obtained through a systematic review of scientific publications on the organism between 2008 to 2017. Literature was very heterogeneous, had inappropriate study designs, unrepresentative sample size, and different diagnostic methods. Lack of data on the required variables and other factors reduced the number of studies to be included in the final meta-analysis substantially. The final systematic review and meta-analyses were done in 30 studies.

According to this review, the first published result that reported *Escherichia coli* in foods of animal origin was in 2008 (Hiko et al., 2008). However, from that year onwards an incredible effort has been noted to report the organism's occurrence in different foods. Studies increased these days regarding number and quality by applying the latest diagnostic techniques like latex agglutination and molecular (PCR) methods. The other qualities of studies were sampling not only the food but also working environment like swabs of workers hand, knife and handling equipment. Theses sampling approaches are appreciated because they are helpful in identifying sources of contamination.

The overall pooled prevalence of *Escherichia coli* was 15% in all samples across Ethiopia means a relatively higher level of occurrence (Ajayi et al., 2011). This organism is responsible for severe infection in Ethiopia (Ayana et al., 2015). Based on the result of this systematic review and meta-analysis, one can argue that the source of the diseases can be contaminated foods from an animal origin. Traditionally in Ethiopia, it is costmary to consume raw meat like *birndo, dulet* and *kitfo* (Avery, 2004). High prevalence level of the organism in these foodstuffs, and raw meat consumption habit can be factors responsible for infections occurred in the country. That is why we suggest raw milk and meat consumption habit of Ethiopians should be prohibited or handled with caution. The overall prevalence of the organism in meat was 16% meaning raw meat consumption may result in contracting *Escherichia coli* infections. In milk, the overall pooled prevalence result was 26%. This high level of occurrence can be attributed to contamination of milk with cattle feces during milking because cattle are the reservoirs of the organism. In addition to feces, poor personal hygiene, contaminated equipment can also be suspected as a source of contamination. However, the occurrence level of *Escherichia coli* in environmental samples was not significant in this systematic review and meta-analysis. This result can be an indication of the need for further studies involving other environmental samples to predict where we should act to reduce its level of occurrence.

Most of the eligible studies retrieved were conducted in central Ethiopia. This can be due to most of milk and meat processing plants are found in this area which can attract the attention of researchers and funders in investigating the occurrence of the organism. Even though this approach may not be wrong at all, it is wise to involve a broader area of research as the condition can be worth in rural households where poor hygienic practice and raw food consumption is imminent. According to studies included, the overall pooled prevalence of the organism was lower in central Ethiopia (9% with a 95% confidence interval of 7%–11%). This can be due to most studies conducted in this area might apply advanced diagnostic techniques that avoid false positives and also a relatively higher level of hygienic practice may be in place. Central Ethiopia includes the capital Addis Ababa which is relatively civilized in applying hygienic measures in food processing plants, and farms than other parts of the country.

Based on diagnostic techniques used, the prevalence of the organism was lower in molecular diagnostic technique followed by a latex agglutination test (Table 1). However, it was higher in biochemical staining techniques. This can be attributed to biochemical test may only estimate at species level while advanced diagnosis techniques can identify the pathogenic strain. These methods should be further confirmed by more advanced methods of examination to estimate STEC.

One of the reasons for the higher level of occurrence of the organism in foods of animal origin can be poor diagnosis method used. Most of the studies included in this review did not identify pathogenic strains like *Escherichia coli* O157: H7. This can be attributed to poor infrastructure in most research institutes and Universities to implement advanced techniques. However recently, the application of a latex agglutination test has increased according to studies included in this review. Based on the molecular and serological test, the overall pooled prevalence of pathogenic *E. coli* (*Escherichia coli* O157) was found 4% in all samples. This level of prevalence might be low. However, the organism is responsible for severe infections that occurred in the country (Disassa et al., 2017; Mersha et al., 2010). For that matter, a comprehensive investigation of the organism with advanced detection methods should be in place.

As indicated in the result section, the between-study variability was high. This variation can be due to different studies involving a different method of diagnosis, different sample size, different study locations and many more reasons. The result of meta-regression indicated that effect size estimates were significantly predicted by a diagnostic method used, sample size and study year reported. This is an indication of expecting higher variation between studies.

## 5 Conclusion

This systematic review and meta-analysis showed that the level of *Escherichia coli* in foods of animal origin is high. This is a clear message that planning of mitigation strategies to reduce the impact of this organism is mandatory. This should be done sooner than later not only to protect exposed group but also to compete in the international market of animal products. If the country wants to achieve the long-awaited membership to world trade organization, it needs to reduce the occurrence of this and other foodborne organisms to an acceptable level. The pathogenic strain *Escherichia coli* O157: H7 was reported only in a few studies. This is because of diagnostic techniques used that are not capable of identifying at the strain level. This and other known pathogenic strains should be well studied by applying modern methods of diagnosis to identify as well as to estimate the actual status in foods of animal origin precisely. Besides, future studies should bear in mind to include rural households of the country where the level of hygienic practices is expected to be low.

## Declarations

### Author contribution statement

Ayalew Assefa: Conceived and designed the experiments; Analyzed and interpreted the data; Wrote the paper.

Amare Bihon: Performed the experiments; Wrote the paper.

### Funding statement

This research did not receive any specific grant from funding agencies in the public, commercial, or not-for-profit sectors.

### Competing interest statement

The authors declare no conflict of interest.

### Additional information

No additional information is available for this paper.
